# IL-23 in arthritic and inflammatory pain development in mice

**DOI:** 10.1186/s13075-020-02212-0

**Published:** 2020-07-07

**Authors:** Kevin M.-C. Lee, Zihao Zhang, Adrian Achuthan, Andrew J. Fleetwood, Julia E. Smith, John A. Hamilton, Andrew D. Cook

**Affiliations:** 1 Department of Medicine, Royal Melbourne Hospital, The University of Melbourne, Parkville, Victoria 3050 Australia; 2 Adaptive Immunity, GSK Medicines Research Centre, Stevenage, Hertfordshire, UK; 3 Australian Institute for Musculoskeletal Science (AIMSS), The University of Melbourne and Western Health, St. Albans, Victoria Australia

**Keywords:** IL-23, GM-CSF, TNF, CCL17, Arthritis, Pain

## Abstract

**Background:**

The cytokine, interleukin-23 (IL-23), can be critical for the progression of inflammatory diseases, including arthritis, and is often associated with T lymphocyte biology. We previously showed that certain lymphocyte-independent, inflammatory arthritis and pain models have a similar requirement for tumour necrosis factor (TNF), granulocyte macrophage-colony stimulating factor (GM-CSF), and C-C motif ligand 17 (CCL17). Given this correlation in cytokine requirements, we explored whether IL-23 might interact with this cytokine cluster in the control of arthritic and inflammatory pain.

**Methods:**

The role of IL-23 in the development of pain-like behaviour was investigated using mouse arthritis models (zymosan-induced arthritis and GM-CSF-, TNF-, and CCL17-driven monoarticular arthritis) and inflammatory pain models (intraplantar zymosan, GM-CSF, TNF, and CCL17). Additionally, IL-23-induced inflammatory pain was measured in *GM-CSF*^*−/−*^, *Tnf*^*−/−*^, and *Ccl17*^*E/E*^ mice and in the presence of indomethacin. Pain-like behaviour and arthritis were assessed by relative weight distribution in hindlimbs and histology, respectively. Cytokine mRNA expression in knees and paw skin was analysed by quantitative PCR. Blood and synovial cell populations were analysed by flow cytometry.

**Results:**

We report, using *Il23p19*^*−/−*^ mice, that innate immune (zymosan)-driven arthritic pain-like behaviour (herein referred to as pain) was completely dependent upon IL-23; optimal arthritic disease development required IL-23 (*P* < 0.05). Zymosan-induced inflammatory pain was also completely dependent on IL-23. In addition, we found that exogenous TNF-, GM-CSF-, and CCL17-driven arthritic pain, as well as inflammatory pain driven by each of these cytokines, were absent in *Il23p19*^*−/−*^ mice; optimal disease in these mBSA-primed models was dependent on IL-23 (*P* < 0.05). Supporting this cytokine connection, it was found conversely that IL-23 (200 ng) can induce inflammatory pain at 4 h (*P* < 0.0001) with a requirement for each of the other cytokines as well as cyclooxygenase activity.

**Conclusions:**

These findings indicate a role for IL-23 in innate immune-mediated arthritic and inflammatory pain with potential links to TNF, GM-CSF, CCL17, and eicosanoid function.

## Background

Pain is a significant symptom associated with many musculoskeletal conditions, for example, rheumatoid arthritis (RA) and psoriatic arthritis (PsA), leading to functional impairment and poor quality of life. Pro-inflammatory cytokines, in addition to their roles in disease progression in inflamed joints, have been implicated in arthritic pain development, but also generally in pain progression [1, 2]. For example, inhibition of the action of tumour necrosis factor (TNF) [3, 4] or granulocyte macrophage-colony stimulating factor (GM-CSF) [5] ameliorates joint pain in animal models of inflammatory arthritis and also clinically in RA patients [6, 7]. We recently described a new GM-CSF➔CCL17 pathway in monocytes/macrophages in vitro, which we found to be important in vivo not only for inflammatory arthritic pain and disease development [8–10], but also for osteoarthritic pain and disease [10]. This pathway could also be relevant to TNF biology due to the potential interdependence between the actions of TNF and GM-CSF [9].

The cytokines, IL-23 and IL-17, have important roles in the pathogenesis of animal models of inflammation, including arthritis, and some human chronic inflammatory diseases [11, 12]. IL-23 is a member of the IL-12 family and consists of both an IL-23-specific p19 subunit and a p40 subunit which is shared with IL-12 [13]. IL-23 is known to stimulate the development of Th17 cells and the production of IL-17 [14]. This IL-23/IL-17 axis has been implicated in the development of autoimmune/inflammatory diseases, such as PsA [12]. The common view is that IL-23 is intimately linked with Th cell biology with IL-17 being under the control of IL-23 [15–18]. However, clinical trial data indicate that they can be uncoupled indicating broader biologies [11]. In preclinical models, mice deficient in IL-23p19 were fully protected from antigen-induced arthritis (AIA) [19], collagen-induced arthritis (CIA) [20, 21], and experimental allergic encephalomyelitis (EAE) [22]; IL-23 was also required for the induction of joint inflammatory mediators including TNF [20]. There is additional evidence that IL-23 and TNF expression can be interdependent including in arthritis patients [23–26]. However, little is known about the role(s) of IL-23 in pain development.

Links between IL-23 and GM-CSF in macrophages and dendritic cells (DCs) have been noted [27–30], and IL-23-dependent secretion of GM-CSF by Th cells has been shown to be crucial in EAE development [15, 16]. A positive feedback loop has been proposed in which the GM-CSF produced by Th cells may further induce IL-23 production by antigen-presenting cells [15, 30, 31]. An additional loop between these cytokines has been suggested in intestinal inflammation, involving group 3 innate lymphoid cells (ILC3s) and myeloid populations [32, 33].

The GM-CSF➔CCL17 pathway, as well as its potential linkage to TNF, can regulate pain and arthritic disease in lymphocyte-independent models [8, 9]. Given the above background data, we decided to explore whether there might be a new IL-23 biology in the area of inflammatory and arthritic pain which may link with the actions of GM-CSF, TNF, and CCL17 in this context and which would not necessarily involve T lymphocytes. In the present study, using mostly *Il23p19*-deficient (*Il23p19*^*−/−*^) mice, we demonstrate that similar to TNF, GM-CSF, and CCL17, IL-23 is also required for the development of zymosan-induced arthritis (ZIA) and its associated inflammatory pain-like behaviour (herein referred to as pain). We also show that IL-23 is required for GM-CSF-, TNF-, and CCL17-driven arthritic pain and disease, as well as for pain induced by intraplantar (i.pl.) administration of these cytokines. Furthermore, we found that IL-23 itself can induce inflammatory pain which is in turn dependent on these cytokines and on cyclooxygenase activity.

## Methods

### Mice

The following mice were used: *GM-CSF (Csf2)*^−/−^ (Ludwig Institute for Cancer Research) [34], *Tnf*^*−/−*^ (The Walter and Eliza Hall Institute (WEHI), Parkville, Australia) [35], *Ccl17*^*E/E*^ (in which both copies of *Ccl17* have been replaced by enhanced green fluorescent protein (EGFP)) (from I. Förster) [36], and *Il23p19*^*−/−*^ mice (from M. Smyth) [37]. All gene-deficient mice were backcrossed onto the C57BL/6 background (WEHI) for more than 10 generations. A total of 408 mice were used in this study. Mice were fed standard rodent chow and water ad libitum. Sex- and age-matched mice were used; experiments were approved by the University of Melbourne Animal Ethics Committee and the GSK Policy on the Care, Welfare and Treatment of Animals.

### Zymosan-induced arthritis model

For the induction of the zymosan-induced arthritis (ZIA) model [8, 9, 38, 39], mice were injected with 300 μg of sonicated zymosan (Sigma-Aldrich) in a 10-μl volume into the left knee joint, while the contralateral knee received saline as a control. On day 7, arthritic joints were collected for gene expression and histologic analysis.

### Inflammatory pain models

Pain was induced by intraplantar (i.pl.) injection (10 μl) of either zymosan (100 μg, Sigma-Aldrich), mouse TNF (20 ng, R&D Systems), mouse GM-CSF (20 ng, Peprotech), mouse CCL17 (50 ng, Biolegend) [8, 9], mouse IL-23 (50, 100, and 200 ng, R&D Systems), or saline into the left hind footpad. Paw swelling was measured using spring callipers (Mitutoyo, Tokyo, Japan). For blocking cyclooxygenase activity, indomethacin (12.5 μg/paw) was injected at *t* = 0.

### mBSA-induced arthritis models

Monoarticular arthritis was induced as before [5, 8, 40] by intra-articular (i.a.) injection of 100 μg mBSA in 10 μl saline into the right knee on day 0, the left knee being injected with saline, followed by a s.c. injection, in the scruff of the neck on days 0–2, of either mouse GM-CSF (500 ng, R&D Systems), mouse TNF (500 ng, R&D Systems), mouse CCL17 (600 ng, R&D Systems), or saline. Mice were sacrificed (day 7), and knee joints collected for histology [8, 9].

For monoclonal antibody (mAb) administration, mice were given 150 μg anti-IL-23p19 (G23-8, eBiosceience™) or isotype (IgG1) control. For prophylactic administration of mAb, mice were i.p. injected on days − 2 and 0; for therapeutic administration of mAb, mice were i.p. injected after pain was evident (i.e. days 1 and 4 for mBSA/TNF and mBSA/GM-CSF models, respectively).

### Assessment of pain-like behaviour—incapacitance meter

As an indicator of pain-like behaviour, the differential distribution of weight over a 3-s period between the inflamed hindlimb relative to the non-inflamed hindlimb was measured using an incapacitance meter (IITC Life Science Inc., USA). This technique has been validated for measurement of both arthritic knee and footpad pain [8–10, 38, 41]. Mice were acclimatized to the incapacitance meter on at least three occasions prior to the commencement of the experiment. Three measurements were taken for each time point and averaged.

The value of weight distribution was calculated as a relative pressure on the left hindlimb to the contralateral hindlimb and expressed as a percentage by the formula:

$$ \Delta  \mathrm{W}=\frac{\mathrm{wL}}{\mathrm{wR}}\times 100\% $$

where wL and wR are the pressure reading of the left and right hindlimb, respectively.

### Histology

At termination, the knee joints were removed, fixed, decalcified, and paraffin embedded [5, 8, 42]. Frontal sections (7 μm) were stained with H&E*.* For ZIA, cell infiltration, proteoglycan loss (Safranin O/Fast Green stain), and bone erosions were scored separately from 0 (normal) to 3 (severe) as before [8, 39]. For the mBSA/TNF, mBSA/GM-CSF, and mBSA/CCL17 models, cellular infiltration, synovitis (synovial hyperplasia), pannus formation, cartilage damage, and bone erosion were scored separately from 0 (normal) to 5 (severe) as described previously [5, 8, 43]. Briefly, soft tissue inflammation, assessed in the infrapatellar fat pad, the joint capsule, and the area adjacent to the periosteal sheath, was graded according to the extent of cellular infiltration and angiogenesis. Synovitis (synovial hyperplasia) was defined as hyperplasia of the synovium, but did not include pannus formation. Pannus was defined as hypertrophic synovial tissue forming a tight junction with the articular surface. Evaluation of cartilage and bone damage was based on loss of cartilage matrix, disruption and loss of cartilage surface, and the extent and depth of the subchondral bone erosion. Total histologic score was calculated as the sum of scores with a maximal score of 25. Scoring was done blindly by two independent researchers and compared for consistency. Results shown are from one researcher.

### Quantitative PCR

qPCR experiments were performed as described previously [8–10, 38]. Briefly, total RNA was extracted using Isolate II RNA Mini Kit (Bioline, Taunton, MA) and reverse transcribed using Tetro reverse transcriptase (Bioline, Taunton, MA). Quantitative PCR was carried out using QuantStudio™ 5 (Applied Biosystems™, Carlsbad, CA) and pre-developed TaqMan probe/primer combinations for murine *Il23p19*, *Tnf*, *Csf2*, *Ccl17*, and *Ubc* (Life Technologies). All samples were assayed in duplicate. Threshold cycle numbers were transformed to ΔCt values, and the results were expressed relative to reference gene, *Ubc* [8–10, 38]*.*

### Cell population analysis

Blood and joint cells were collected as previously described [10, 38, 44], then analysed by flow cytometry. Briefly, Fc receptors on cells were blocked with normal mouse serum (1/4 dilution) and stained with fluorochrome-conjugated mAbs specific for mouse CD45-PerCP (clone 30-F11), CD115-APC (clone AFS98), CD11b-APC-Cy7 (clone M1/70), Ly6G-PE-Cy7 (clone 1A8), F4/80-BV421 (clone BM8), and the corresponding isotype controls, either from BD Biosciences, Biolegend, or eBiosciences. Cells were analysed using a CytoFLEX LX (Beckman Coulter).

### Statistics

For pain readings and gene expression, a two-way ANOVA was used; for histologic scores, the Mann-Whitney two-sample rank test and a two-way ANOVA were used. For population analysis, Student’s *t* test was used (GraphPad Software, version 5.04, San Diego, CA). A Bonferroni post hoc test was used when appropriate. Data are pooled from two experiments. Data were plotted as mean ± SEM with significance *P* values as indicated. A *P* value less than 0.05 was considered significant.

## Results

### IL-23 is required for zymosan-induced arthritic pain and optimal disease development as well as for zymosan-induced inflammatory pain

#### Arthritis

**Fig. 1:**
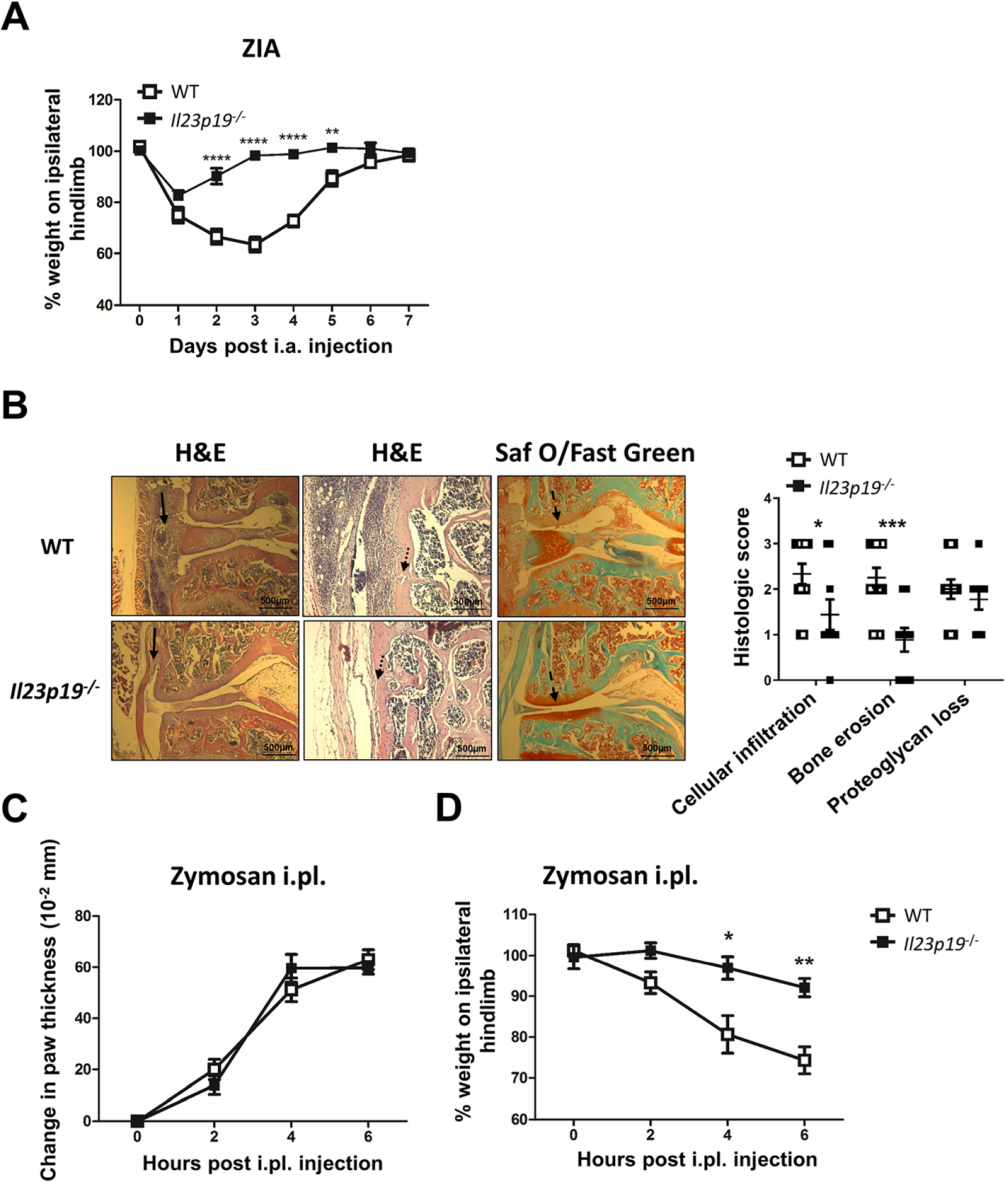
IL-23 is required for zymosan-induced arthritic pain and optimal disease development as well as for zymosan-induced inflammatory pain. **a**, **b** WT and *Il23p19*^*−/−*^ mice received an intra-articular (i.a.) injection of zymosan. **a** Pain (incapacitance meter [ratio of weight bearing on injected relative to non-injected hindlimb]; a value less 100 indicates pain) and **b** arthritis (histology: H&E stain, Safranin O/Fast Green stain, day 7) were measured. Arrows indicate the following features, respectively: solid arrows, cellular infiltration; dotted arrows, bone erosion; and dashed arrows, proteoglycan loss. **c**, **d** WT and *Il23p19*^*−/−*^ mice received an intraplantar (i.pl.) injection of zymosan. **c** Paw swelling (× 10^−2^ mm) and **d** pain (incapacitance meter) were measured over a 6-h period. Data are expressed as mean ± SEM; **a**–**d** WT and *Il23p19*^*−/−*^ male mice (saline, *n* = 10; zymosan, *n* = 10). For statistical analysis, a two-way ANOVA for pain and a Mann-Whitney test for histology were used. *P < 0.05, **P < 0.01, ***P < 0.01, ****P < 0.0001, WT vs. *Il23p19*^*−/−*^ mice

IL-23 plays a role in the disease progression of adaptive immune-driven arthritis models, for example, the AIA [19] and CIA models [20, 21]. We examined if it is also important for both arthritic pain and disease development in an innate immune-driven model. ZIA, induced by an intra-articular (i.a.) injection of zymosan, is a widely used macrophage-dependent [45], monoarticular arthritis model; we have previously reported that endogenous GM-CSF, TNF, and CCL17 are required for pain and optimal arthritis development in this model [8, 9]. Following i.a. zymosan administration, WT mice developed pain-like behaviour (herein referred to as pain) by a change in weight distribution (incapacitance meter) (see the “Methods” section), which was evident until day 5 [38]. In contrast, *Il23p19*^*−/−*^ mice did not (Fig. 1a), indicating IL-23 dependence. Histologically, *Il23p19*^*−/−*^ mice developed significantly less ZIA than WT mice, as assessed by cell infiltration (*P* = 0.04, 95% confidence interval (CI) 0.032, 1.746) and bone erosion (*P* = 0.0008, 95% CI 0.504, 2.218) (Fig. 1b).

The similar ZIA data for *Il23p19*^−/−^ mice and for GM-CSF, TNF, and CCL17 gene-deficient mice [8, 9] suggest that IL-23 function might be linked with each of these cytokines in this model. To begin to examine these possibilities, the IL-23 dependence for the gene expression of the other cytokines was measured in the ZIA joints using WT and *Il23p19*^−/−^ mice. It can be seen that zymosan-stimulated mRNA levels (day 7) for *Csf2* (gene for GM-CSF) and *Ccl17*, but not for *Tnf*, demonstrated IL-23 dependence (Additional file 1: Figure S1A); conversely, zymosan-stimulated *Il23p19* mRNA expression in the joint was reduced in *GM-CSF*^*−/−*^ and *Tnf*^*−/−*^ mice, but not in *Ccl17*^*E/E*^ mice, when compared to the value in WT mice (Additional file 1: Figure S1B), indicating a dependence on the first two cytokines (see the “Discussion” section).

#### Inflammatory pain

Intraplantar (i.pl.) zymosan administration is a well-studied inflammatory pain model [8, 9, 38, 46, 47]. We tested whether IL-23 was also required in this model. At 4 and 6 h post-i.pl. zymosan, paw swelling (increased thickness) was evident to the same extent in WT and *Il23p19*^*−/−*^ mice (Fig. 1c); however, in contrast, pain was only evident in WT mice (Fig. 1d), again indicating IL-23 dependence.

These data indicate that IL-23 is required for zymosan-induced arthritic and inflammatory pain and optimal zymosan-induced arthritis development.

### IL-23 is required for the GM-CSF-, TNF-, and CCL17-driven arthritic pain and disease development

To explore further whether IL-23 might be linked to GM-CSF, TNF, and/or CCL17 in the control of arthritic pain and disease, we again utilized arthritis models which are driven by each of these cytokines individually in a lymphocyte-independent manner [5, 8, 9]—these other monoarticular arthritis models all involve systemic administration of a cytokine into a mouse with a methylated BSA (mBSA) “primed” joint and are a convenient approach to explore potential pathways downstream of a particular cytokine [8, 9]. These models are as follows: GM-CSF-driven (i.a. mBSA day 0, subcutaneous (s.c.) GM-CSF days 0–2, [5]), TNF-driven (i.a. mBSA day 0, s.c. TNF days 0–2, [9]), and CCL17-driven (i.a. mBSA day 0, s.c. CCL17 days 0–2, [8]) arthritis. It should be noted, however, that administration of exogenous systemic cytokine may not necessarily inform about the role of the endogenous molecule [48–50].

**Fig. 2:**
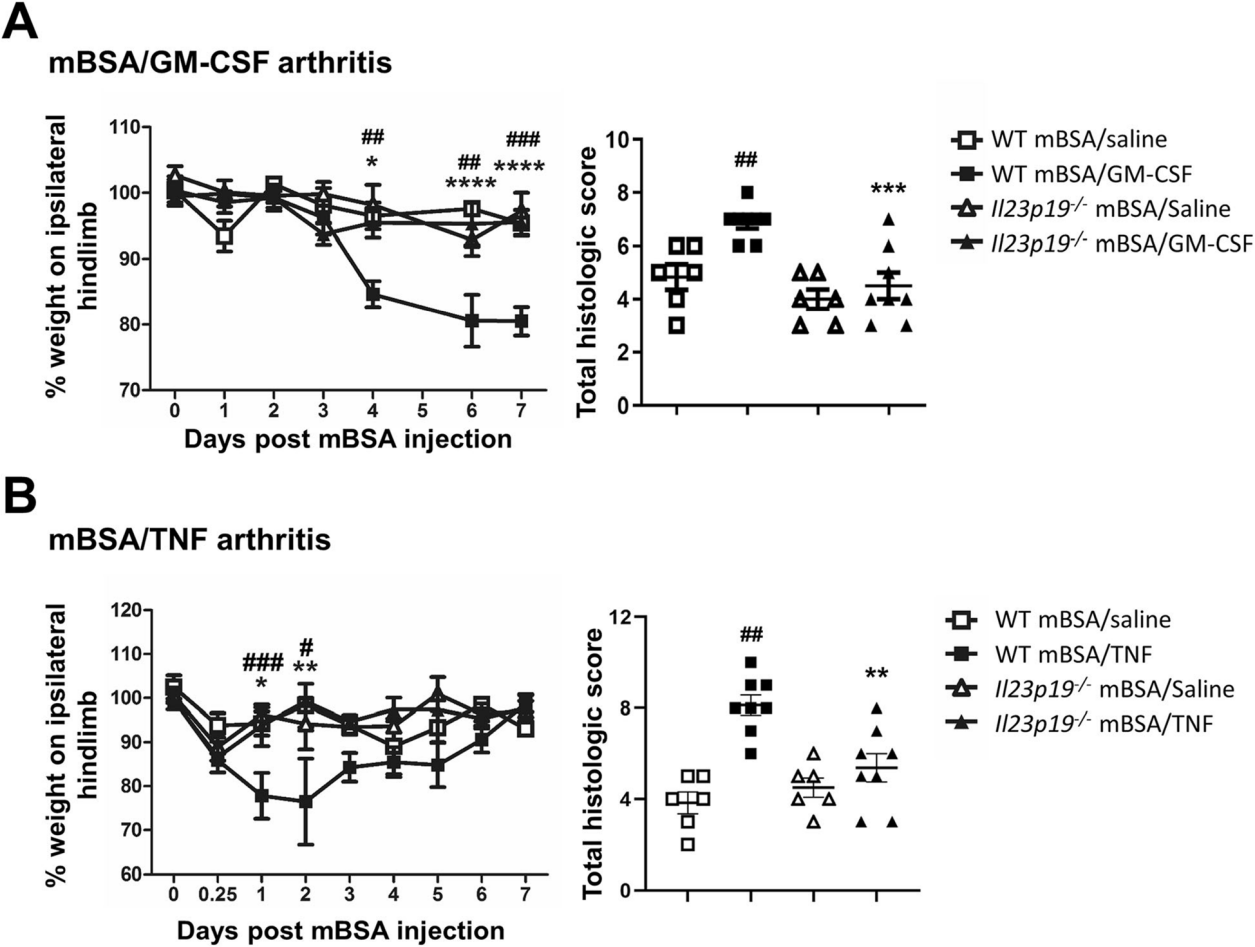
IL-23 is required for GM-CSF- and TNF-driven arthritic pain and disease development. **a** mBSA/GM-CSF arthritis (mBSA intra-articular (i.a.) [day 0]; GM-CSF or saline subcutaneous (s.c.) [day 0–2]) and **b** mBSA/TNF arthritis (mBSA i.a. [day 0]; TNF or saline s.c. [day 0–2]) were induced in WT and *Il23p19*^*−/−*^ mice. Pain (incapacitance meter) and arthritis (histology, day 7) were measured. Data are expressed as mean ± SEM; **a**, **b** WT and *Il23p19*^*−/−*^ male mice (mBSA/saline, *n* = 6; mBSA/GM-CSF and mBSA/TNF, *n* = 8). For statistical analysis, a two-way ANOVA was used. ^#^*P* < 0.05, ^##^*P* < 0.01, ^###^*P* < 0.001, WT saline vs. WT GM-CSF or TNF. *P < 0.05, **P < 0.01, ***P < 0.001, ****P < 0.0001, WT GM-CSF or TNF vs. *Il23p19*^*−/−*^ GM-CSF or TNF

**Fig. 3:**
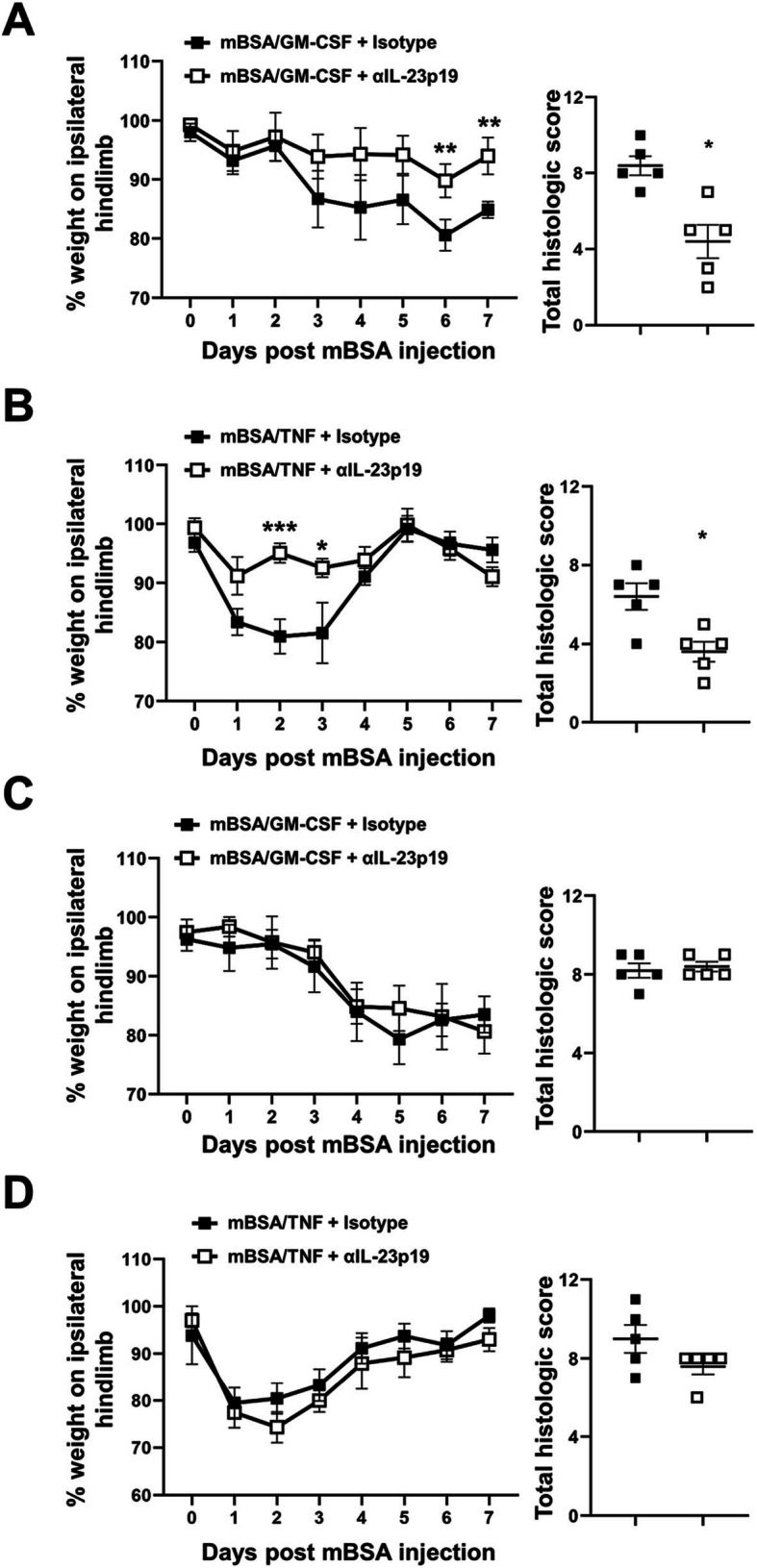
IL-23 is required for the onset of GM-CSF- and TNF-driven arthritic pain and disease development. mBSA/GM-CSF (mBSA intra-articular (i.a.) [day 0]; GM-CSF or saline subcutaneous (s.c.) [day 0–2]) and mBSA/TNF (mBSA i.a. [day 0]; TNF or saline s.c. [day 0–2]) arthritis were induced in WT mice. The mice were treated i.p. with anti-IL-23p19 mAb or IgG1 isotype control, either **a**, **b** prophylactically (150 μg, days − 2 and 0) or **c**, **d** therapeutically (150 μg, on day 4 or day 1) for mBSA/GM-CSF and mBSA/TNF, respectively. Pain (incapacitance meter) and arthritis (histology, day 7) were assessed. Data are expressed as mean ± SEM; **a**, **c** WT mBSA/GM-CSF male mice (IgG1 isotype control, *n* = 5; anti-IL-23p19 mAb, *n* = 5), **b**, **d** WT mBSA/TNF male mice (IgG1 isotype control, *n* = 5; anti-IL-23p19 mAb, *n* = 5). For statistical analysis, a two-way ANOVA was used. *P < 0.05, **P < 0.01, ***P < 0.001, isotype vs. anti-IL-23p19 mAb

Following induction of mBSA/GM-CSF (Fig. 2a) and mBSA/TNF (Fig. 2b) arthritis in WT mice, as expected, pain developed by days 4 (*P* = 0.0081, 95% CI − 21.93, − 1.782) and 1 (*P* = 0.0007, 95% CI − 26.97, − 5.902), respectively [5, 9], which is not seen in the s.c. saline control group; arthritic pain was, however, not seen in *Il23p19*^*−/−*^ mice in either of these models (Fig. 2a, b), indicating an IL-23 dependence. Histologically, *Il23p19*^*−/−*^ mice were also protected from GM-CSF- (*P* = 0.0004, 95% CI 1.094, 3.656) and TNF-driven (*P* = 0.001, 95% CI 1.116, 4.384) arthritis development (Fig. 2a, b). We have previously shown that GM-CSF is required throughout the mBSA/TNF arthritis model, whereas TNF is only required in the early initiation phase of the mBSA/GM-CSF model [9]. In order to determine when IL-23 was required in these models, we again used a mAb approach administering an anti-IL-23p19 mAb or isotype control, either prophylactically (on days − 2 and 0) or therapeutically (when pain is evident—for mBSA/GM-CSF, day 4; for mBSA/TNF, day 1). Following prophylactic administration of anti-IL-23p19 mAb, GM-CSF- and TNF-driven arthritic pain development were prevented (Fig. 3a, b, respectively); prophylactic anti-IL-23p19 mAb also reduced disease development in both models (GM-CSF-driven model, *P* = 0.0159; TNF-driven model, *P* = 0.0317) (Fig. 3a, b). On the other hand, therapeutic anti-IL-23p19 mAb treatment was unable to ameliorate the GM-CSF- and TNF-driven arthritic pain or disease (Fig. 3c, d).

Seeing that the arthritic joint in both of these cytokine-driven arthritis models is injected with mBSA and that the IL-23 requirement appears to be at the initiation stage, we considered that this timing could be linked to IL-23-dependent cell population changes occurring in the mBSA “primed” joint prior to systemic cytokine administration, i.e. the IL-23 might not in fact be downstream of exogenous cytokine action per se. To begin to test this proposal, synovial macrophage (CD45^+^CD11b^+^F4/80^+^Ly6G^−^) and neutrophil (CD45^+^CD11b^+^Ly6G^+^) numbers were analysed (flow cytometry) in naïve and mBSA-injected joints from WT and *Il23p19*^*−/−*^ mice (Additional file 2: Figure S2A). While no differences in the cell numbers in naïve joints were evident (Additional file 2: Figure S2B), significantly fewer macrophages, but not neutrophils, were present in *Il23p19*^*−/−*^ joints compared to WT joints 1 day following i.a. mBSA injection (Additional file 2: Figure S2C). Consistent with these findings in the joints, there were fewer steady-state blood monocytes (CD11b^+^CD115^+^) present in *Il23p19*^*−/−*^ compared to WT mice, but similar numbers of blood neutrophils (CD11b^+^Ly6G^+^CD115^−^) (Additional file 2: Figure S2D-E). The difference in macrophage number in the mBSA “primed” joints of WT and *Il23p19*^*−/−*^ mice could therefore be contributing to the differences in pain and arthritis (mainly synovitis) noted upon the administration of exogenous (s.c.) cytokines (Fig. 2a, b).

**Fig. 4:**
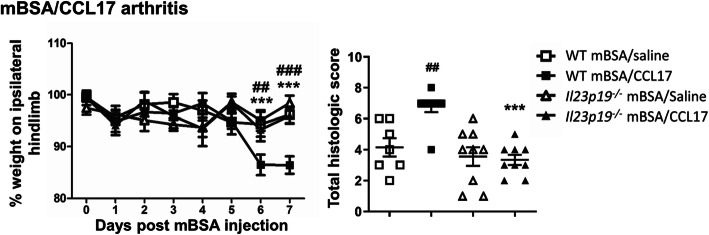
IL-23 is required for CCL17-driven arthritic pain and disease development. mBSA/CCL17 arthritis (mBSA i.a. [day 0]; CCL17 or saline s.c. [day 0–2]) was induced in WT and *Il23p19*^*−/−*^ mice. Pain (incapacitance meter) and arthritis (histology, day 7) were measured. Data are expressed as mean ± SEM; WT female mice (mBSA/saline, *n* = 8; mBSA/CCL17, *n* = 8), *Il23p19*^*−/−*^ female mice (mBSA/saline, *n* = 8; mBSA/CCL17, *n* = 9). For statistical analysis, a two-way ANOVA was used. ^##^*P* < 0.01, ^###^*P* < 0.001, WT saline vs. WT CCL17. ***P < 0.001, WT CCL17 vs. *Il23p19*^*−/−*^ CCL17

For mBSA/CCL17 arthritis in WT mice, as expected, pain developed by day 6 [8]. Even though TNF and GM-CSF are not required in this model [9], interestingly, arthritic pain was again not seen in *Il23p19*^*−/−*^ mice (Fig. 4); as measured histologically, *Il23p19*^*−/−*^ mice were also protected from CCL17-driven arthritis development (*P* = 0.0002, 95% CI 1.501, 5.332).

Collectively, these data indicate that IL-23 is required in some way for GM-CSF-, TNF-, and CCL17-induced arthritis pain and disease development.

### IL-23 is required for GM-CSF-, TNF-, and CCL17-driven inflammatory pain

**Fig. 5:**
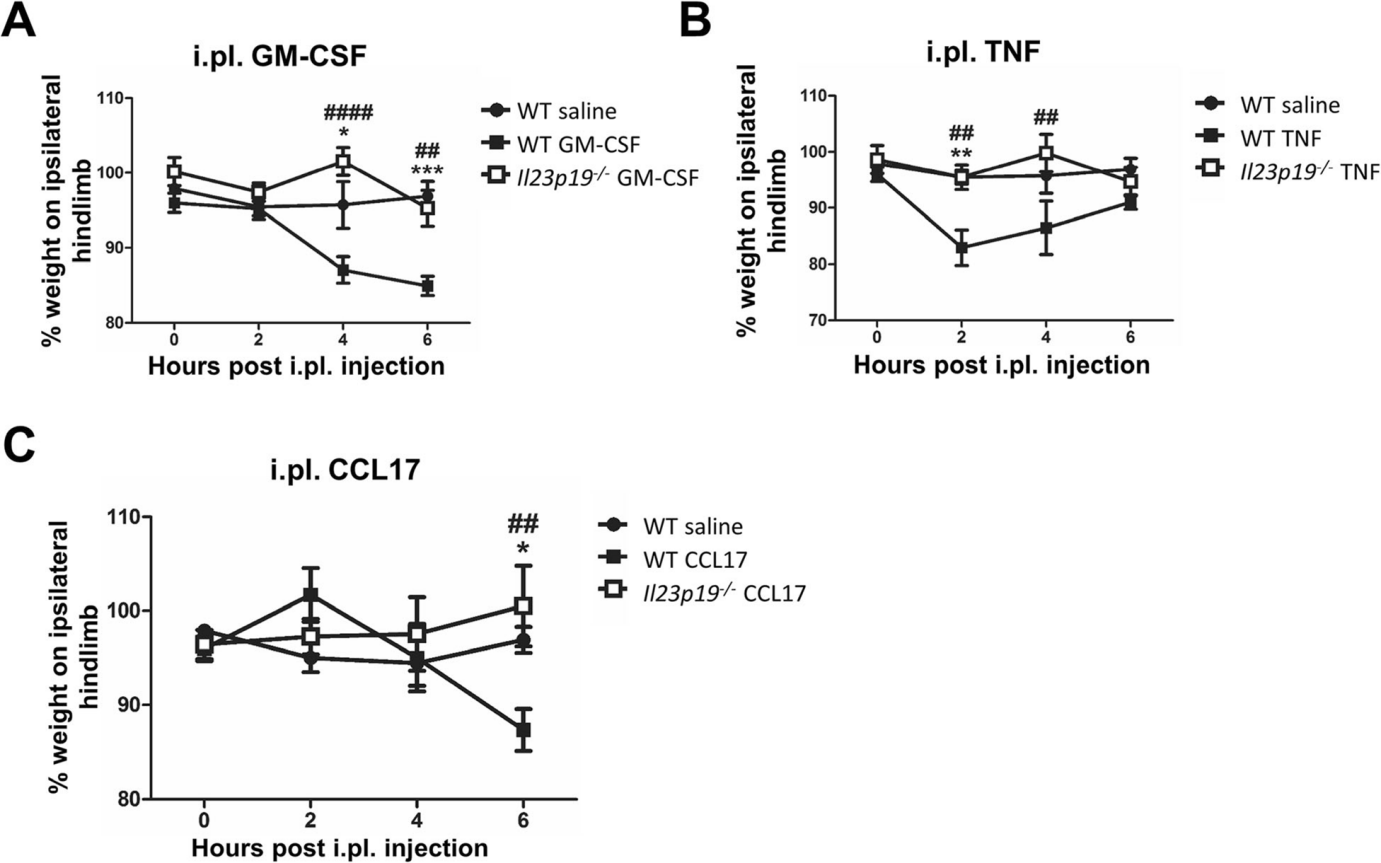
IL-23 is required for GM-CSF-, TNF-, and CCL17-driven inflammatory pain. WT and *Il23p19*^*−/−*^ mice received an intra-plantar (i.pl.) injection of saline, **a** GM-CSF, **b** TNF, or **c** CCL17. Pain (incapacitance meter) was measured. Data are expressed as mean ± SEM; **a**–**c** WT male mice (saline, *n* = 8; GM-CSF/TNF/CCL17, *n* = 10), *Il23p19*^*−/−*^ male mice (GM-CSF/TNF, *n* = 10; CCL17, *n* = 15). For statistical analysis, a two-way ANOVA was used. ^##^*P* < 0.01, ^####^*P* < 0.0001, WT saline vs. WT GM-CSF, TNF, or CCL17. *P < 0.05, **P < 0.01, ***P < 0.001, WT GM-CSF, TNF, or CCL17 vs. *Il23p19*^*−/−*^ GM-CSF, TNF, or CCL17, respectively

We next explored IL-23 involvement in the GM-CSF-, TNF-, and CCL17-driven acute inflammatory pain models [5, 8, 9]—we have previously used these models to explore how these respective cytokines can elicit inflammatory pain and how they might be linked in this context [5, 8, 9]. As before [5, 8, 9], at the particular dose used, WT mice injected i.pl. with GM-CSF (Fig. 5a), TNF (Fig. 5b), and CCL17 (Fig. 5c) exhibited pain at around 4 h (*P* < 0.0001, WT saline vs. WT GM-CSF), 2 h (*P* = 0.0078, WT saline vs. WT TNF), and 6 h (*P* = 0.0058, WT saline vs. WT CCL17) post-injection, respectively. As for the corresponding cytokine-driven arthritis pain models (Figs. 2 and 4), *Il23p19*^*−/−*^ mice were protected from the pain development in each of these inflammatory pain models (Fig. 5a–c), indicating again an IL-23 dependence.

These data indicate that IL-23 is also required for the development of inflammatory pain driven by each of these cytokines in the mouse paw.

### IL-23 induces pain with a requirement for GM-CSF, TNF, CCL17, and cyclooxygenase activity

**Fig. 6:**
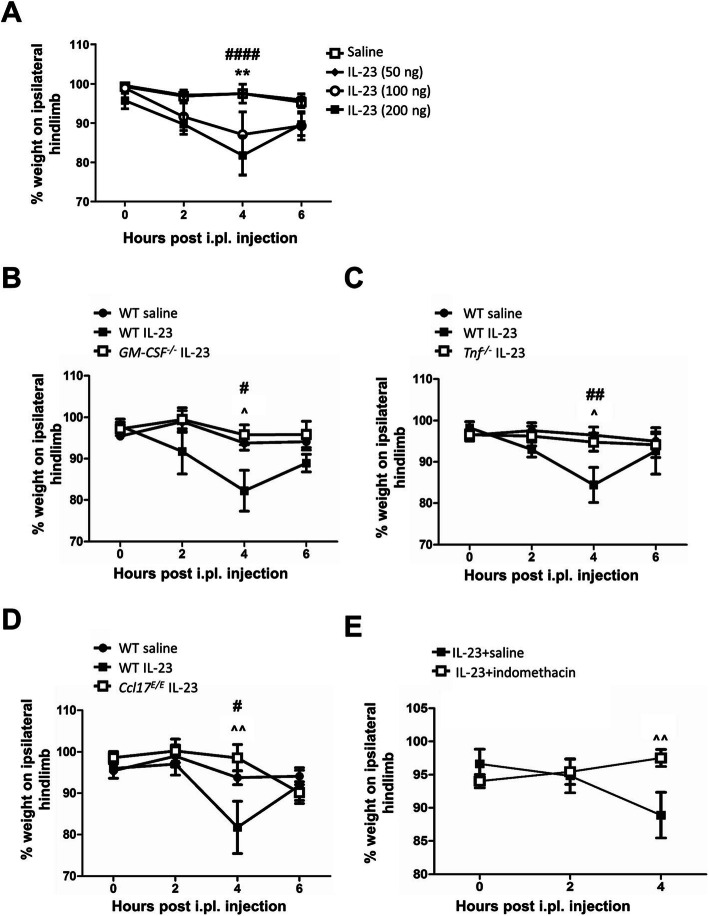
IL-23 induces pain with a requirement for GM-CSF, TNF, CCL17, and cyclooxygenase activity. **a** WT mice received an intraplantar (i.pl.) injection of saline or IL-23 (50 ng, 100 ng, or 200 ng). Pain (incapacitance meter) was measured. **b**–**d** An i.pl. injection of saline or IL-23 (200 ng) was given to **b** WT and *GM-CSF*^*−/−*^ mice, **c** WT and *Tnf*^*−/−*^ mice, and **d** WT and *Ccl17*^*E/E*^ mice. Pain was measured. **e** WT mice received i.pl. IL-23 (200 ng) with or without indomethacin (12.5 μg/paw i.pl. at *t* = 0) on day 0. Pain was measured. Data are expressed as mean ± SEM; **a** WT female mice (saline, *n* = 5; 50 ng, *n* = 10; 100 ng, *n* = 10; 200 ng, *n* = 15), **b**–**d** WT, *GM-CSF*^*−/−*^, *Tnf*^*−/−*^, and *Ccl17*^*E/E*^ male mice (saline, *n* = 6; IL-23, *n* = 10), **e** WT female mice (saline, *n* = 10; indomethacin, *n* = 10). For statistical analysis, a two-way ANOVA was used. **P < 0.01, saline vs. IL-23 (100 ng). ^#^*P* < 0.05, ^##^*P* < 0.01, ^####^*P* < 0.0001, saline vs. IL-23 (200 ng). ^*P* < 0.05, ^^*P* < 0.01, WT IL-23 vs. *GM-CSF*^*−/−*^ IL-23, *Tnf*^*−/−*^ IL-23, or *Ccl17*^*E/E*^ IL-23, respectively; IL-23 + saline vs. IL-23 + indomethacin

Having shown above that IL-23 is required for TNF-, GM-CSF-, and CCL17-driven pain, we hypothesized that IL-23 alone might be sufficient to induce pain and perhaps with the involvement of the other cytokines. To begin to test these possibilities, WT mice received increasing doses of i.pl. IL-23. 100 and 200 ng, but not 50 ng, of IL-23 induced an acute pain within 4 h (*P* = 0.0041, saline vs. 100 ng; *P* < 0.0001, saline vs. 200 ng) (Fig. 6a). To determine whether the other cytokines were required, we injected IL-23 (200 ng) i.pl. into *GM-CSF*^*−/−*^, *Tnf*^*−/−*^, and *Ccl17*^*E/E*^ mice, respectively—while WT mice exhibited pain (Fig. 6b–d), *GM-CSF*^*−/−*^ (Fig. 6b), *Tnf*^*−/−*^ (Fig. 6c), and *Ccl17*^*E/E*^ (Fig. 6d) mice did not. Since it is known that the pain induced by i.pl. GM-CSF, TNF, and CCL17 requires cyclooxygenase (COX) activity to develop [5, 8, 9], we next determined whether inhibiting COX activity would also suppress IL-23-induced pain. Treating mice with indomethacin suppressed IL-23-induced pain (Fig. 6e), indicating that COX activity, presumably leading to the formation of an eicosanoid (e.g. PGE_2_), is required.

Collectively, these data suggest that exogenous IL-23 induces skin pain via a pathway requiring TNF, GM-CSF, CCL17, and COX activity.

## Discussion

Much IL-23 biology is often associated with that of T lymphocytes in inflammation/auto-immunity (see, for example, [15–18]). The data above provide the first evidence in preclinical models for the involvement of IL-23 in arthritic and inflammatory pain. It should be noted that this involvement occurs independently of lymphocytes since the arthritis models studied do not require them [8, 9]. We previously reported that ZIA pain and disease, as well as zymosan-induced inflammatory pain, were dependent on GM-CSF, TNF, CCL17, and COX activity [8, 9]. We have shown here that IL-23 is also required, indicating at least a correlation, but also suggesting that there might be a link between IL-23 and these mediators in these models. mRNA expression analysis in the ZIA joints examining both IL-23p19 dependence and expression indicated links with the expression of TNF, GM-CSF, and/or CCL17 (Additional file 1: Figure S1), which in turn have themselves been linked in this model [8, 9]. However, more information is needed, for example, on cell population numbers and specific cellular expression in the inflamed ZIA joints of the gene deficient mouse strains studied if these possible connections are to be clarified further.

We have also shown, at least by an approach utilizing models incorporating exogenous cytokines and again without a lymphocyte requirement, that both TNF- and GM-CSF-driven arthritic pain and disease, in addition to requiring each other [8, 9], also require IL-23. In other words, with this approach, IL-23, TNF, and GM-CSF actions can be integrated in the control of arthritic pain and disease even without lymphocyte involvement. In support of these observations, there have been reports associating at least some aspects of IL-23 and TNF biology in vivo in mice and in arthritis patients [22–26, 51, 52]; IL-23 and GM-CSF have also been associated at the level of expression in vivo and in the mutual control of both adaptive and innate immune models of inflammatory/autoimmune disease in mice [15, 16, 30–33, 53–56].

We found previously that CCL17 can also be a critical downstream mediator for both TNF- and GM-CSF-driven arthritic pain and disease progression [8, 9]. However, CCL17-driven arthritic pain and disease, a model again not requiring lymphocytes [8], did not require TNF or GM-CSF [8, 9]—intriguingly, in contrast, we found above that these CCL17-driven responses in the mBSA “primed” joint require IL-23. Therefore, at least for the cytokine-driven arthritis models and, as mentioned, for the ZIA model, IL-23 can be linked with other three cytokines. Of possible mechanistic relevance, in addition to its original definition as a chemokine for T cell trafficking, CCL17, via its receptor CCR4, has been implicated in DC migration and function [57–59], including a proposed regulation of IL-23 formation via a GM-CSF-dependent pathway [57, 58].

Even though the mBSA/cytokine models are convenient ones, enabling the potential identification of candidate downstream mediators of the algesic and arthritogenic actions of cytokines, it should be borne in mind that they are two stage models, involving systemic administration of a cytokine into a mBSA “primed” joint. The mBSA “priming” stage may contribute to subsequent cytokine effects, as suggested, for example, by our data in Additional file 1: Figure S2, wherein reduced tissue macrophage number might contribute to the lower tissue inflammatory response noted in *Il23p19*^*−/−*^ mice and which in turn could be due to their reduced number of steady-state blood monocytes, as shown before in a bacterial infection model [60].

IL-23 has previously been found to be important for disease progression in preclinical arthritis models. In AIA, IL-23 was required for the development of optimal disease [19]; for CIA, *Il23p19*^*−/−*^ mice were completely protected from arthritis, but similar to our findings above with the mBSA/GM-CSF and mBSA/TNF models, mAb inhibition of IL-23 after disease onset was ineffective [20, 21]. However, both the AIA and CIA models are associated with an adaptive immune response [61, 62], but interestingly, in the context of our findings above, they are also TNF and GM-CSF dependent [41, 63–65], with the AIA model in addition being CCL17 dependent [8]. We have previously examined the cellular composition of ZIA joints (day 7) and shown significant macrophage and neutrophil infiltration [38]. Studies have implicated IL-23 in the regulation of the number of inflammatory macrophages [60], as mentioned, as well as neutrophils [23, 66, 67], while in AIA, IL-23 drives neutrophil migration into the synovial cavity in a prostaglandin-dependent manner [66]. IL-23 biology has also been linked to that of granulocyte-CSF (G-CSF) [68], the latter cytokine contributing to neutrophil number and function in inflammation, including in ZIA pain and disease development [38]. Therefore, the reduced cellular infiltration seen above in *Il23p19*^*−/−*^ mice in the arthritis models studied above could possibly be due to impaired IL-23-induced monocyte and neutrophil migration [60, 66] with an impact subsequently on the degree of pain and joint structure modification. We also found that the arthritis data above were similar between male and female mice. We acknowledge that while measuring relative changes in weight distribution of the hindlimbs as an indicator of arthritic pain is a highly relevant and well-established method [5, 8–10, 38], it does not fully capture the pain phenotype.

Recently, a new IL-12 family member was discovered in mice, namely IL-39, which is a heterodimer consisting of IL-23p19 (shared with IL-23) and EBI3 (shared with IL-27 and IL-35) [69, 70]. Even though the evidence for human IL-39 remains controversial [71], of possible relevance to our studies, it has been reported that murine IL-39 can activate neutrophils and mediate inflammation in lupus-like disease in mice [69, 70]. Whether IL-39 is contributing to our findings above is unknown.

We also demonstrated that IL-23 could itself induce pain in the paw, which was dependent on the other cytokines, in line with the connection noted in the inflamed joints. Of possible relevance to these observations, IL-23 and CCL17 can induce skin inflammation, the former via TNF [24, 72], and both are implicated clinically in psoriasis and atopic dermatitis [73, 74]. Another connection between IL-23 and the other three cytokines would also appear to be that IL-23-induced inflammatory pain was dependent on COX activity, mostly likely COX-2, since COX-2 inhibitors suppressed inflammatory pain caused by TNF, GM-CSF, and CCL17 [8, 9]. COX-2-derived products, such as PGE_2_, have been widely linked to IL-23 formation and function, both in in vitro and in vivo [66, 75–79]. In addition to PGE_2_, nociceptive neuron-produced CGRP has also been shown to induce the formation of IL-23 via cutaneous dendritic cells [67]. This finding suggests that there might not be a simple linear sequence of cytokine production but multiple mediator loops contributing as highlighted before [9].

## Conclusion

We have noted previously the critical involvement of a GM-CSF➔CCL17 pathway in the regulation of experimental arthritic and inflammatory pain, which can also be linked to the action of TNF and eicosanoid(s) [8, 9]. Importantly, the GM-CSF➔CCL17 pathway appears to be active in RA patients [80]. Intriguingly, we now provide evidence that IL-23 should also be considered as possibly being linked to this pathway and can exhibit a lymphocyte-independent biology in some pathologies. The literature evidence for the involvement of IL-23 in pain progression is limited [81] although an anti-IL-23p19 mAb can reduce pain in PsA patients [82]. Of possible relevance to our findings with IL-23 in the paw, it has been reported that nociceptive sensory neurons drive IL-23-mediated psoriasiform skin inflammation [67]. Given our data above, it would seem that further research on the role of IL-23 in the regulation of pain would be worthwhile.

## Supplementary information

**Additional file 1: Figure S1.** Dependence of *Csf2*, *Tnf*, *Ccl17* and *Il23p19* mRNA expression in zymosan-induced arthritis joints. WT, *Il23p19*^*-/-*^, *GM-CSF*^*-/-*^, *Tnf*^*-/-*^ and *Ccl17*^*E/E*^ mice received an intra-articular (i.a.) injection of saline or zymosan. Joint mRNA expression (day 7) of (A) *Csf2*, *Tnf*, *Ccl17* and (B) *Il23p19* was analyzed. Data are expressed as mean ± SEM; (A-B) WT, *Il23p19*^*-/-*^, *GM-CSF*^*-/-*^, *Tnf*^*-/-*^ and *Ccl17*^*E/E*^ female mice (saline *n*=5, zymosan *n*=8). For statistical analysis, a two-way ANOVA was used. #*p*<0.05, ##*p*<0.01, ###*p*<0.001, saline vs. zymosan. *p<0.05, **p<0.01, ***p<0.001, WT vs. *Il23p19*^*-/-*^, *GM-CSF*^*-/-*^ or *Tnf*^*-/-*^ mice, respectively.

**Additional file 2: Figure S2.** IL-23 is required for optimal mBSA-induced synovial macrophage response. (A-C) Analysis of neutrophils and macrophages from naïve and mBSA-injected joints (day 1) of WT and *Il23p19*^*-/-*^ mice. (A) Representative FACS plots showing the gating strategy used to identify synovial neutrophils (CD11b^+^ Ly6G^+^) (R1) and macrophages (CD11b^+^ F4/80^+^) (R2); (B-C) numbers of total cells (CD45^+^), neutrophils (R1) and macrophages (R2) in (B) naïve and (C) mBSA-injected joints (day 1) from WT and *Il23p19*^*-/-*^ mice. (D-E) Analysis of blood monocytes and neutrophils from naïve WT and *Il23p19*^*-/-*^ mice. (D) Representative FACS plots showing the gating strategy used to identify monocytes (CD11b^+^ CD115^+^) (R3) and neutrophils (CD11b^+^ Ly6G^+^) (R4); (E) numbers of total blood leukocytes, monocytes (R3) and neutrophils (R4). Data are expressed as mean ± SEM; (B-C) WT and *Il23p19*^*-/-*^ female mice (saline/mBSA *n*=10), (E) WT and *Il23p19*^*-/-*^ female mice (*n*=6). For statistical analysis, an unpaired Student’s t-test was used. *p<0.05, WT vs. *Il23p19*^*-/-*^ mice.

## Data Availability

Not applicable
