# Supplementary figures and images for: IL-23 in arthritic and inflammatory pain development in mice
Source: Arthritis Res Ther. 2020 Jul 7;22:123. doi: 10.1186/s13075-020-02212-0 (PMC7345543; doi:10.1186/s13075-020-02212-0)

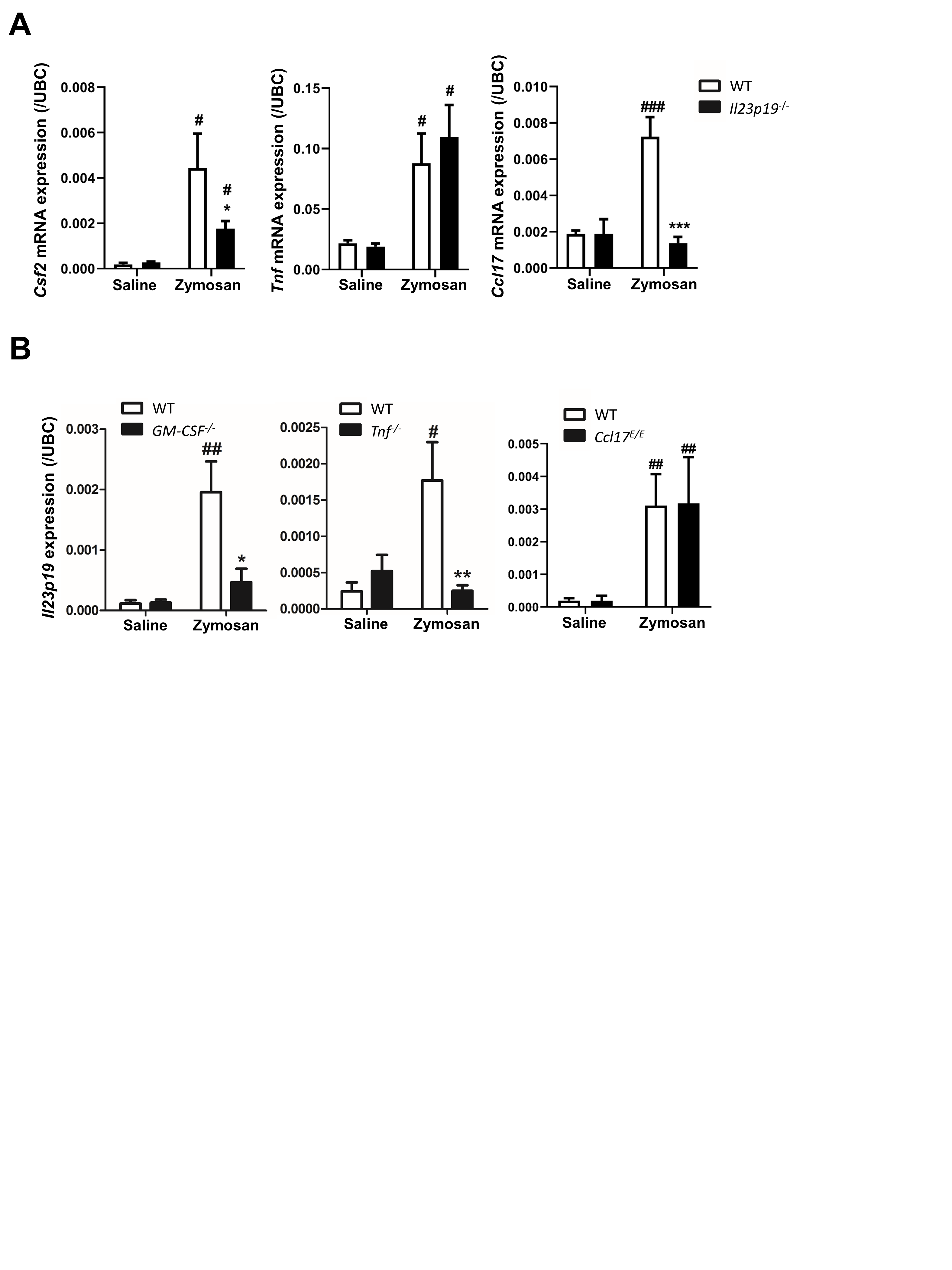

Supplement: Supplementary file 1 — Additional file 1: Figure S1. Dependence of Csf2, Tnf, Ccl17 and Il23p19 mRNA expression in zymosan-induced arthritis joints. WT, Il23p19-/-, GM-CSF-/-, Tnf-/- and Ccl17E/E mice received an intra-articular (i.a.) injection of saline or zymosan. Joint mRNA expression (day 7) of (A) Csf2, Tnf, Ccl17 and (B) Il23p19 was analyzed. Data are expressed as mean ± SEM; (A-B) WT, Il23p19-/-, GM-CSF-/-, Tnf-/- and Ccl17E/E female mice (saline n=5, zymosan n=8). For statistical analysis, a two-way ANOVA was used. #p<0.05, ##p<0.01, ###p<0.001, saline vs. zymosan. *p<0.05, **p<0.01, ***p<0.001, WT vs. Il23p19-/-, GM-CSF-/- or Tnf-/- mice, respectively. [file 13075_2020_2212_MOESM1_ESM.tif]

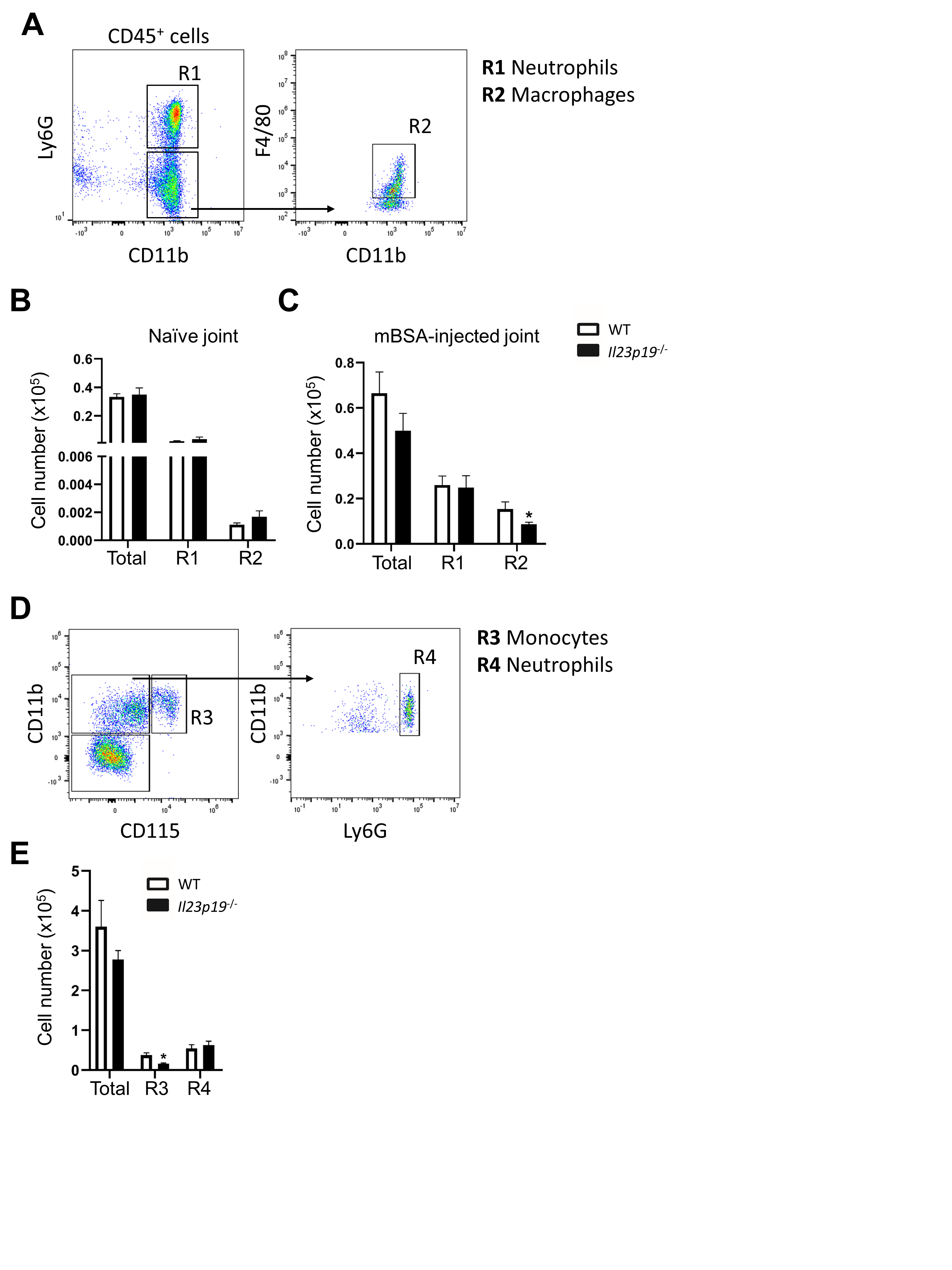

Supplement: Supplementary file 2 — Additional file 2: Figure S2. IL-23 is required for optimal mBSA-induced synovial macrophage response. (A-C) Analysis of neutrophils and macrophages from naïve and mBSA-injected joints (day 1) of WT and Il23p19-/- mice. (A) Representative FACS plots showing the gating strategy used to identify synovial neutrophils (CD11b+ Ly6G+) (R1) and macrophages (CD11b+ F4/80+) (R2); (B-C) numbers of total cells (CD45+), neutrophils (R1) and macrophages (R2) in (B) naïve and (C) mBSA-injected joints (day 1) from WT and Il23p19-/- mice. (D-E) Analysis of blood monocytes and neutrophils from naïve WT and Il23p19-/- mice. (D) Representative FACS plots showing the gating strategy used to identify monocytes (CD11b+ CD115+) (R3) and neutrophils (CD11b+ Ly6G+) (R4); (E) numbers of total blood leukocytes, monocytes (R3) and neutrophils (R4). Data are expressed as mean ± SEM; (B-C) WT and Il23p19-/- female mice (saline/mBSA n=10), (E) WT and Il23p19-/- female mice (n=6). For statistical analysis, an unpaired Student’s t-test was used. *p<0.05, WT vs. Il23p19-/- mice. [file 13075_2020_2212_MOESM2_ESM.tif]
